# The Effect of Vertical and Oblique Inclinations on Fracture Stability and Reoperation Risks in Femoral-Neck Fractures of Nongeriatric Patient

**DOI:** 10.3389/fbioe.2021.782001

**Published:** 2021-11-03

**Authors:** Dajun Jiang, Shi Zhan, Hai Hu, Hongyi Zhu, Changqing Zhang, Weitao Jia

**Affiliations:** Department of Orthopedic Surgery and Orthopedic Biomechanical Laboratory, Shanghai Jiao Tong University Affiliated Sixth People’s Hospital, Shanghai, China

**Keywords:** femoral neck fracture (hip fracture), nongeriatric patients, fracture inclination, fracture stability, reoperation, three-dimensional measurement and analysis

## Abstract

**Background**: For nongeriatric patients with femoral neck fractures (FNFs), preoperative evaluation of fracture three-dimensional inclination is essential to identify fracture stability, select appropriate fixation strategies, and improved clinical prognoses. However, there is lack of evaluation system which takes into account both vertical and oblique inclinations. The purpose of this study was to comprehensively investigate the effect of vertical and oblique inclinations on fracture stability and reoperation risks.

**Methods:** We retrospectively reviewed the medical records of 755 FNFs patients with over 2 years follow-up. The 3-D inclination angle in vertical (α) and oblique plane (β) were measured based on CT images. The optimal threshold for unstable 3-D inclination were identified by seeking the highest Youden Index in predicting reoperation and validated in the biomechanical test. According to the cut-off value proposed in the diagnostic analysis, forty-two bone models were divided into seven groups, and were all fixed with traditional three parallel screws. Interfragmentary motion (IFM) was used for comparison among seven groups. The association between reoperation outcome and 3-D inclination was analysed with a multivariate model.

**Results and Conclusion:** The overall reoperation rate was 13.2%. Unstable 3-D inclination angles with an optimally determined Youden index (0.39) included vertical (*α* > 70°) and oblique (50°<α < 70° and *β* > 20°/*β* < −20°) types. Biomechanical validation showed these fractures had significantly greater (*p* < 0.05) interfragmentary motion (1.374–2.387 mm vs. 0.330–0.681 mm). The reoperation rate in 3-D unstable group (32.7%) is significantly (*p* < 0.001) higher than that in 3-D stable group (7.9%). Multivariate analysis demonstrated that 3-D inclination angle was significantly (OR = 4.699, *p* < 0.001) associated with reoperation. FNFs with *α* > 70°; 50°<*α* < 70° and *β* > 20°/*β* < −20° are real unstable types with significantly worse interfragmentary stability and higher reoperation risks. Fracture inclination in vertical and oblique planes is closely related to reoperation outcomes and may be a useful complement to the way FNFs are currently evaluated.

## Introduction

Femoral neck fractures (FNFs) in patients younger than 60 years old frequently result from high-energy impact and remain a clinical challenge to treat (Slobogean et al., 2015; Anne et al., 2018). Internal fixation treatment is generally preferred over arthroplasty for these patients, but healing complications and reoperations are particularly common (Slobogean et al., 2015; Anne et al., 2018). Avascular necrosis (AVN) rate is most common (14.3%) (Slobogean et al., 2015), followed by rates of 9.3% for non-unions (NU) (Slobogean et al., 2015), and 13.0% reoperation rate (Anne et al., 2018). FNFs in nongeriatric patients are difficult to treat because of the high-energy violent nature of the insult, unfavorable biomechanical environment, tenuous vasculature, and inappropriate treatment strategies. An improved understanding of fracture morphology and the underlying stability is the prerequisite to select an appropriate internal fixation and rehabilitation plan in order to maximally reduce the high risks of complications. However, for years, debate has continued about what the optimal internal fixation is for these patients, a controversy that is exacerbated by a lack of a satisfactory principle to evaluate fracture stability and predict clinical prognosis accurately.

Currently, for nongeriatric patients, Pauwels classification, based on two-dimensional (2-D) X-ray, is a relatively widely used system to evaluate fracture stability and guiding internal fixation choice. Typically, the Pauwels angle reflects vertical inclination in the vertical plane. But recent analysis of fracture morphology (Collinge C A et al., 2014; Sarfani et al., 2021) revealed that the fracture surface in axial oblique plane was not always sagittally oriented. Increasing evidences indicated that fixation stability is influenced by both of the fracture’s vertical and oblique inclinations (Collinge C A et al., 2014; Wright_et al., 2020; Wang et al., 2021), but Pauwels angle is insensitive to the latter important parameter. To date, the effect of axial obliquity on fracture stability and clinical outcome has not been thoroughly studied in all FNF types. Consequently, there is still necessary to draw a more complete picture of fracture stability which takes into account both vertical and oblique inclinations.

With a large scale of nongeriatric patients with all FNF types, the purpose of the current study was to comprehensively describe the 3-D inclination angle in both vertical and oblique planes, and to investigate the combined effect of fracture inclinations in multiple planes on fracture stability and reoperation risks.

## Materials and Methods

### Patient Recruitment

**FIGURE 1:**
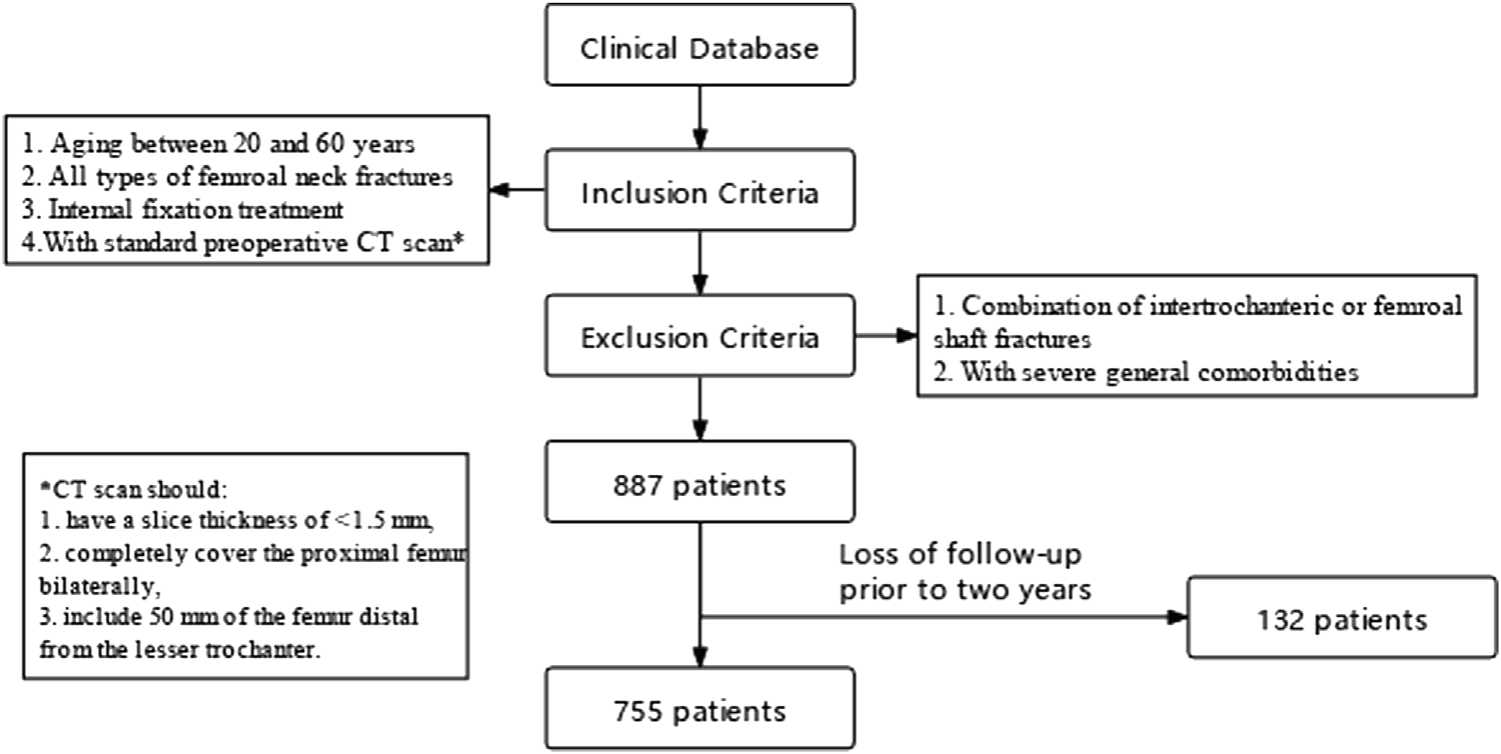
Patient selection process.

From January 2013 to December 2018, we conducted a retrospective search for patients with FNFs in ten orthopaedic wards of one general hospital, which is the second largest medical centre in this country with over 51,000 operations annually. According to our inclusion/exclusion criteria, a convenience sample of 755 patients were finally included in the analysis (Figure 1). All subjects were given written informed consent to participate, and the study was approved by the local institutional ethics review board (No. 2016-143). The essential parameters of each case such as age, gender, smoking history, number of follow-up months, displacement degree, comminution, reduction quality, and fixation strategy were documented as baseline information.

### Radiographic Measurement

**FIGURE 2:**
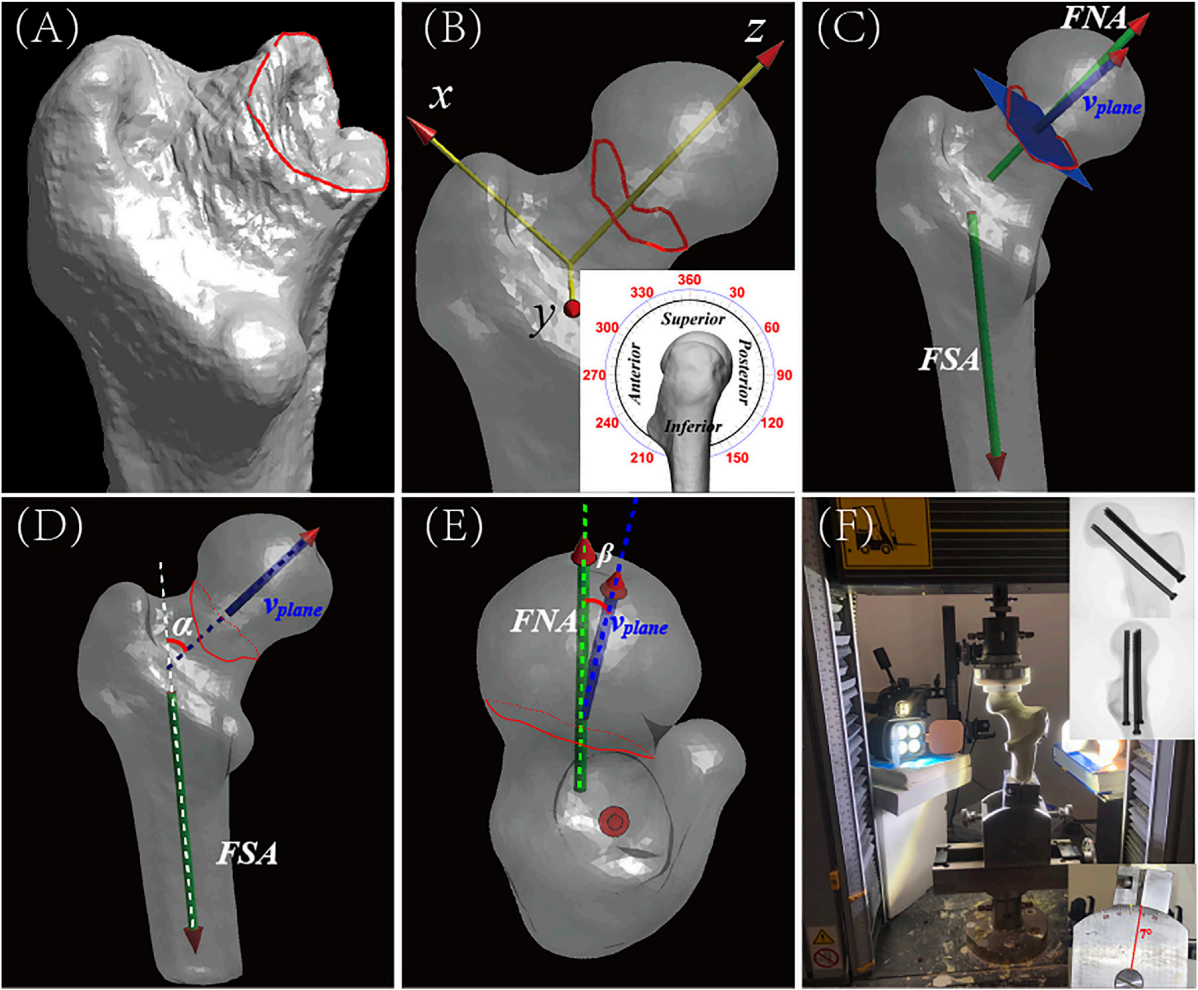
The measurement of Fracture inclination in multiple planes from CT images, and the setup for the biomechanical validation study. **(A)** Anteroposterior (AP) view of reconstructed distal fragment of proximal femur and neck showing position of FNF. The fracture line (red) was manually traced along the cortical edges of the distal fragment in 3-Matics® software. Proximal fragment is hidden in this image. For orientation purposes, lesser trochanter occupies the lower foreground. **(B)** Local coordinate axes (X, Y, Z) overlaid on complete 3-D reconstruction. Local coordinate axes (X, Y, Z) overlaid on complete 3-D reconstruction. Fracture line was divided into equal sections or degrees using the classical clock-face system (inset, lateral view). For orientation, 0°/360° (i.e., 12 o’clock) points toward the superior part of the femoral head and 180° (i.e., 6 o’clock) points toward its inferior part. The clock-face system was then digitally transposed onto the custom proximal femoral coordinate system. **(C)** AP view of reconstructed proximal femur showing orientation of the fracture plane, which is represented by the blue-colored rectangle. v_plane_ represents the normal vector of the fracture plane. FSA, femoral shaft axis; FNA, femoral neck axis. **(D)** AP view of the reconstructed proximal femur showing how proposed classification parameters were determined. *α* (red) is defined as the angle between v_plane_ (blue, projected on vertical plane) and the FSA (white dotted line). **(E)** Axial view of the reconstructed femur showing femoral head and determination of proposed classification parameters. *β* is defined as the angle between v_plane_ (blue, projected on coronal plane) and the FNA (green dotted line). *β* is a measure of the axial obliquity of a fracture. **(F)** Photograph of the biomechanical test setup showing a simulated FNF in a Synbone^®^. In the biomechanical test, a simulated fracture was made in a Synbone^®^, the fracture was fixed with standard cannulated orthopaedic screws, and the femoral shaft was fixed in the Instron testing instrument at a shaft adduction angle of 7° (lower inset photograph). Interfragmentary motion (IFM) was then recorded using a VIC-3D 9 system (Correlated Solutions, Inc.). As progressively greater downward force was applied to the fixed Synbone^®^, high-speed digital images were acquired of the Synbone^®^ and stored for later analysis. The digital images were imported into MATLAB software, and IFM was calculated for each of the seven groups of simulated FNFs. Top inset shows postoperative radiograph of fixation screws in Synbone^®^.

The CT images of the 755 patients were analysed using the novel quantitative analysis system using MIMICS and MATLAB software. Three-dimensional bone models were reconstructed based on their CT scans. Outlines of the fracture were drawn along the cortical edges of distal fragment models (Figure 2A). In order to quantify the 3-D inclination angle, we used a coordinate system based on the contralateral femur for reference (Dolatowski et al.;Dimitriou et al., 2016). This reference coordinate system was mirrored and best-fit aligned with the distal fragment using the iterative closest points (ICP) method (Dimitriou et al., 2016). In the coordinate system, the fracture line was divided equally into 360 points according to the classic clock-face system (Lavigne et al.;Lazaro et al., 2015) (Figure 2B). Although the contour of the fracture line is a complex shape, a representative fracture plane is necessary for calculating the inclination angle. Current morphological studies (Collinge C A et al., 2014) typically have only calculated fracture inclination in just one slice of each CT plane, which cannot accurately describe the overall orientation of a complex fracture line. In order to solve this problem more scientifically and quantitively, a best-fitting plane based on the 360 points on the fracture line was created using the least-squares method, and the normal vector (v_plane_) of the plane was defined as its 3-D orientation (Figure 2C). This method is a classic technique in geology for accurately determining the orientation of a rock fracture surface in 3-D (Feng et al., 2001), a problem that shares the same basic parameter space as accurately determining the orientation of a bone fracture in 3-D. The fracture’s 3-D inclination angle was quantified by measuring the angulation of fracture orientation with femoral shaft axis (FSA) in the coronal plane (*α*, Figure 2D) and that with femoral neck axis (FNA) in axial plane (*β*, Figure 2E).

### Clinical Outcomes

The primary endpoint was relevant reoperation due to the advantage of absolute objectivity, which has been extensively applied in clinical studies (Slobogean et al., 2017; Anne et al., 2018). All complications were recorded as secondary outcomes; these included nonunion, femoral neck shortening (Zlowodzki et al., 2008) (>10 mm), and avascular necrosis.

### Clinical Analysis of 3-D Unstable Inclination Angles

**FIGURE 3:**
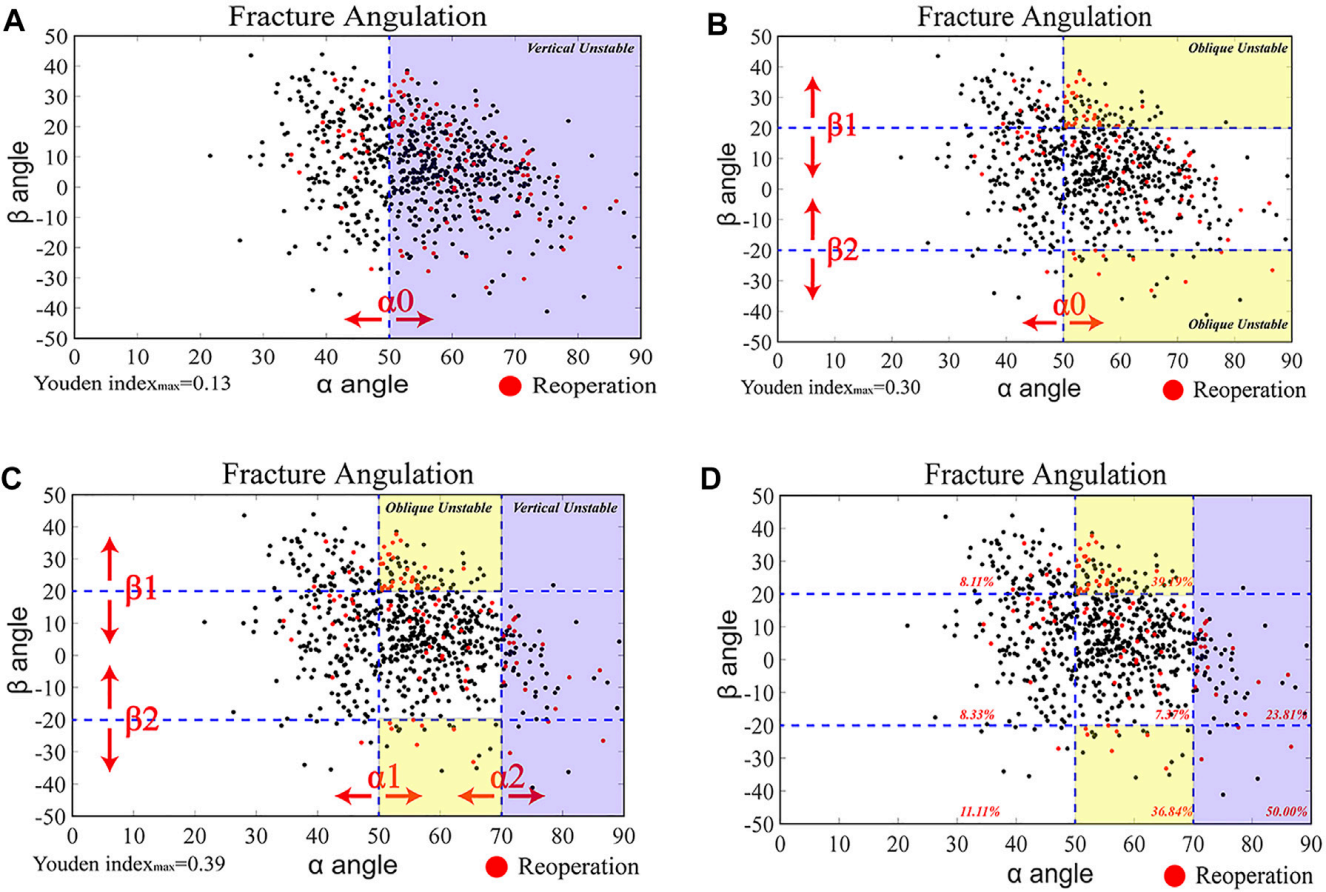
Identification of Three-Dimensional Unstable Inclination angles based on clinical prognosis analyses **(A–C)** Scatterplots of 3-D inclination angles of all FNFs (*n* = 755) (black dots). Reoperated cases (red dots) are overlaid. The identification of 3-D unstable inclination angles was based on the 755 reoperation cases (*n* = 100; See Supplementary Table S2) and their associated *α* and *β* angles calculated from the 3-D reconstructions of their proximal femur and FNFs. Three grouping principles were tested in the diagnostic test: (1) vertical unstable [**(A)**, purple-colored region); (2) oblique unstable [**(B)**, yellow-colored region]; and (3) both vertical and oblique unstable [**(C)**, purple-colored and yellow-colored regions]. **(D)** The optimal threshold values (α0, α1, α2, β1, β2) were identified by calculating the maximum Youden index among over 12,160 possible combinations. The combined vertical (*α* > 70°) and oblique (50°<α < 70° and *β* > 20°/<−20°) unstable types had the highest Youden index (0.39). The reoperation rate percentages (red) are shown in each region.

We identified 3-D unstable inclination angles based on inclination angles and the corresponding reoperation outcome (yes or no, See Supplementary Table S1) for each of the 755 cases. Based on clinical and biomechanical considerations, three grouping principles were evaluated in the “diagnostic test”: 1) only vertical unstable (Figure 3A); 2) only oblique unstable (Figure 3B); and 3) both vertical and oblique unstable (Figure 3C). We calculated the Youden index YI (Habibzadeh et al., 2016) (YI = Specificity + Sensitivity − 1) for all possible combinations of *α* and *β* angles to determine the optimal threshold in predicting reoperation outcome. After identifying the optimal cut-off value, the association between 3-D inclination angles and reoperation outcome was analysed using a multivariant model. Complications and reoperation risks in 3-D unstable groups were compared with those in the stable group.

### Biomechanical Validation

**FIGURE 4:**
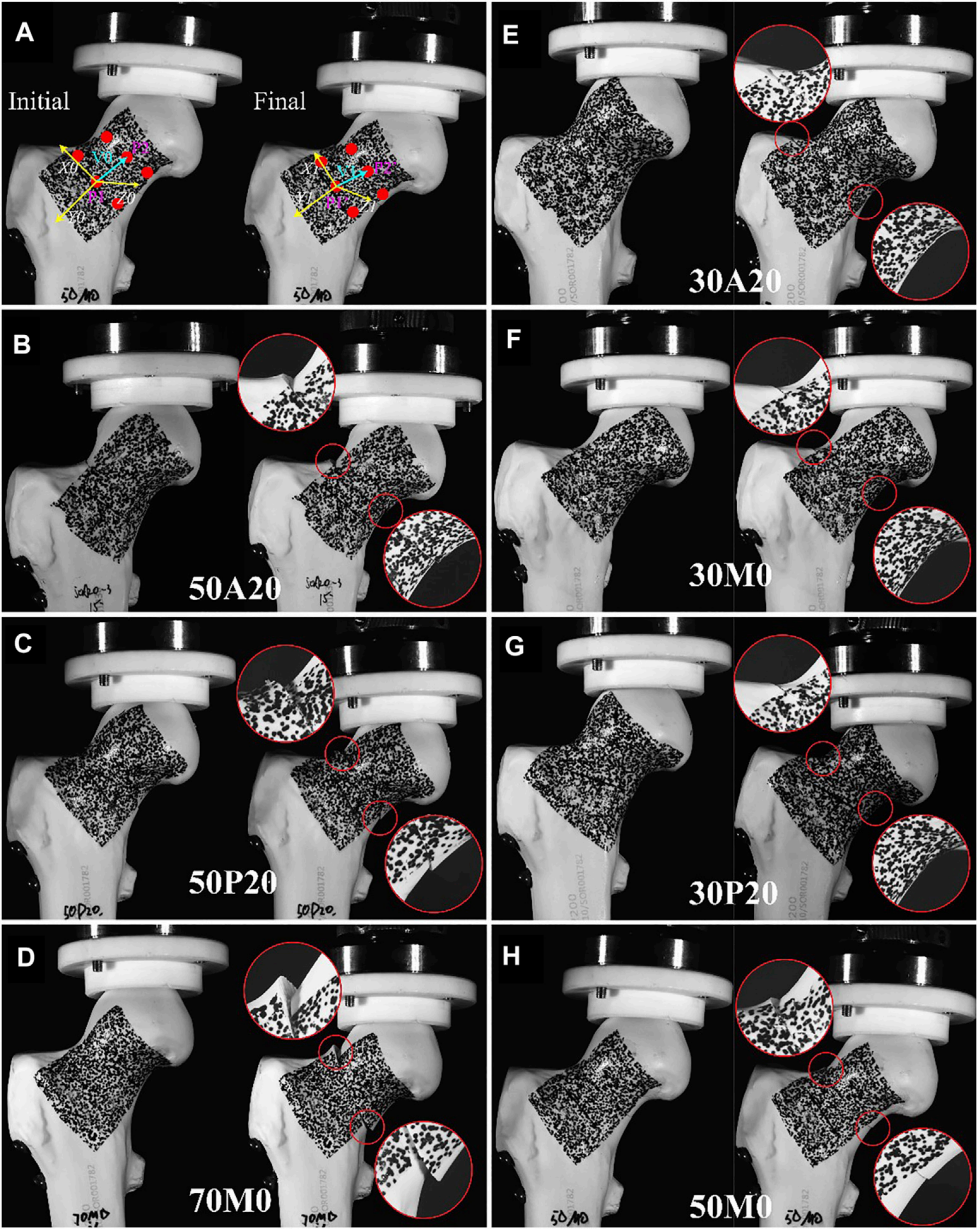
Validation of 3-D unstable inclination angles in a biomechanical test. Illustrating the calculation and results of interfragmentary motion. Interfragmentary motion (IFM) was calculated according to the location of the three paired points (red dotted) in proximal and distal fragments, and the local coordinate system **(A)**. According to the photograph obtained with the VIC-3D, IFM was more severe in unstable groups **(B–D)** compared to stable groups **(E–H)**.

The 3-D unstable fractures were validated by setting up a biomechanical test using commercially available realistic bone simulants. Forty-two Synbone models (model # 2200, Synbone®; Zizers, Switzerland) were divided into seven groups for the biomechanical test. According to the cut-off value proposed in the diagnostic analysis, the fractures plane in each group had the following *α* and β values: 1) 30A20 (*α* = 30°, *β* = -20°); 2) 30M0 (*α* = 30°, *β* = 0°); 3) 30P20 (*α* = 30°, *β* = 20°); 4) 50A20 (*α* = 50°, *β* = -20°); 5) 50M0 (*α* = 50°, *β* = 0°); 6) 50P20 (*α* = 50°, *β* = 20°); and 7) 70M0 (*α* = 70°, *β* = 0°). All fractures were fixed with three cannulated screws positioned in a classic inverted triangle configuration. The 3-D printed guiding plate was applicated to guarantee the accuracy of fracture orientation and screws position (Jiang et al., 2021c). Prior to being mounted on an Instron® system test apparatus, the model bones were speckled with black paint for VIC-3D to identify each spot and document its position during deformation (Figure 4). After that, all models were tested using an Instron load test system, set to a maximum 2000 N vertical loading load angled 7°(Zhang et al., 2018) relative to the FSA (Figure 2F). The main outcome variable for this part of the study was Inter-fragmentary motion (IFM).

IFM was calculated according to the location of the points recorded by the VIC-3D (XR-9M; Correlated Solutions Inc., Irmo, SC, United States). Three paired points from superior, medial, and inferior parts of the femoral neck were selected for analysis. The algorithm used to calculate the IFM was based on vector rather than scalar values. As showed in (Figure 4A), V0 and V1 represent the vectors of two target points at the initial and final state, respectively. Both V0 and V1 were projected into X (V0X, V1X); Y (V0X, V1X); and Z (V0X, V1X) axes (Figure 4A). The two coordinate systems, X0Y0Z0 and X1Y1Z1, were constant in relation to the distal fragment. IFM was calculated based on the formula below. The mean IFM of the three paired nodes was defined as the IFM of the fracture model.

$$\mathrm{IFM}=\sqrt[{2}]{|\underset{V1X}{\to }-\underset{V0X}{\to }{|}^{2}+|\underset{V1Y}{\to }-\underset{V0Y}{\to }{|}^{2}+|\underset{V1Z}{\to }-\underset{V0Z}{\to }{|}^{2}}.$$ (1)

### Statistical Statistics

All calculations were conducted using SAS software (version 9.4 for Windows; SAS Institute, Cary, NC, United States). Categorical variables were compared using the chi-squared test, expressing as frequencies. Continuous variables are presented as means and standard deviations, and were checked for normality firstly using Kolmogorov-Smirnov test. Multiple-group comparisons of parametric continuous variables in the biomechanical validation test were performed using one-way ANOVA. The possible relationship between 3-D inclination angles with reoperation risks was evaluated using a binary logistic regression model. The 3-D inclination angles were regarded as exposure variables and was converted into a binary variable (stable = 0; unstable = 1). Other possible confounding parameters documented as baseline information were also entered as variables into the regression models. Statistical significance was set at *p* < 0.05.

## Results

From January 2013 through the end of December 2018, a total of 755 patients with a mean age of 47.14 ± 10.68 years was included in this study as participants (Table 1). The mean values for *α*, *β* were, respectively, 54.91° ± 10.91°, 6.96° ± 14.65° with ICCs (95% CI) values of 0.78 (0.75–0.80), 0.85 (0.82–0.87).

**TABLE 1:** Demographic and selected clinical characteristics of the study population.

Variable	Total	Reoperation	OR (95% CI)	*p* Value
Participants	755	100 (13.3%)	—	—
Age	47.14 ± 10.68	46.00 ± 10.85	0.988 (0.966–1.010)	0.281
Sex				
Male	410	57 (13.9%)	1	—
Female	345	43 (12.5%)	1.321 (0.760–2.296)	0.324
Smokers	210	29 (13.8%)	1.109 (0.614–2.003)	0.731
Follow-up time	47.89 ± 18.84	50.34 ± 19.25	1.007 (0.995–1.019)	0.256
Displacement degree^c^				
Non-displaced	201	7 (3.5%)	1	—
Displaced	554	93 (16.8%)	3.774 (1.667–8.544)	0.001^b^
Comminution				
No	333	32 (9.60%)	1	—
Yes	422	68 (20.2%)	1.599 (0.979–2.610)	0.061
Reduction quality^d^				
Excellent-Good	639	72 (11.3%)	1	—
Fair-Poor	116	28 (24.1%)	2.117 (1.233–3.635)	0.007^b^
Fixation strategy^e^				
3 cannulated screws	611	69 (11.3%)	1	—
4 cannulated screws	108	20 (18.5%)	1.547 (0.846–2.829)	0.156
Other	36	11 (30.6%)	2.500 (1.096–5.704)	0.029^b^
3-D inclination Angle^f^				
Stable	593	47 (7.9%)	1	0
Unstable	162	53 (32.7%)	4.699 (2.949–7.486)	<0.001^b^

a CI, confidence interval.

b Significant.

c Displacement degree is evaluated by Garden classification.

d Reduction quality was assessed according to the method of Haidukewych and colleagues.

e Other fixation strategies included 32 dynamic hip screws and four buttress plate technique.

f 3-D unstable inclination angles were identified in the current study and were defined as α > 70°; 50° < α < 70° and β > 20° or < −20°.

Among the included patients, 100 (13.2%) cases underwent relevant reoperation for the reasons of non-union (*n* = 12, 7 with arthroplasty, 4 with re-osteosynthesis, 1 with vascularized fibular grafting), avascular necrosis (n = 67, 58 with arthroplasty, 9 with vascularized fibular grafting) and screw withdrawal or protruding (*n* = 21, Removal of implants).

Among the 12,160 possible combinations of cut-off values, 3-D unstable inclination angles with an optimal Youden index (0.39) were identified in fractures having an *α* > 70° (vertical) and 50° < *α* < 70° and *β* > 20°/< -20° (oblique) (Figure 3D).

**FIGURE 5:**
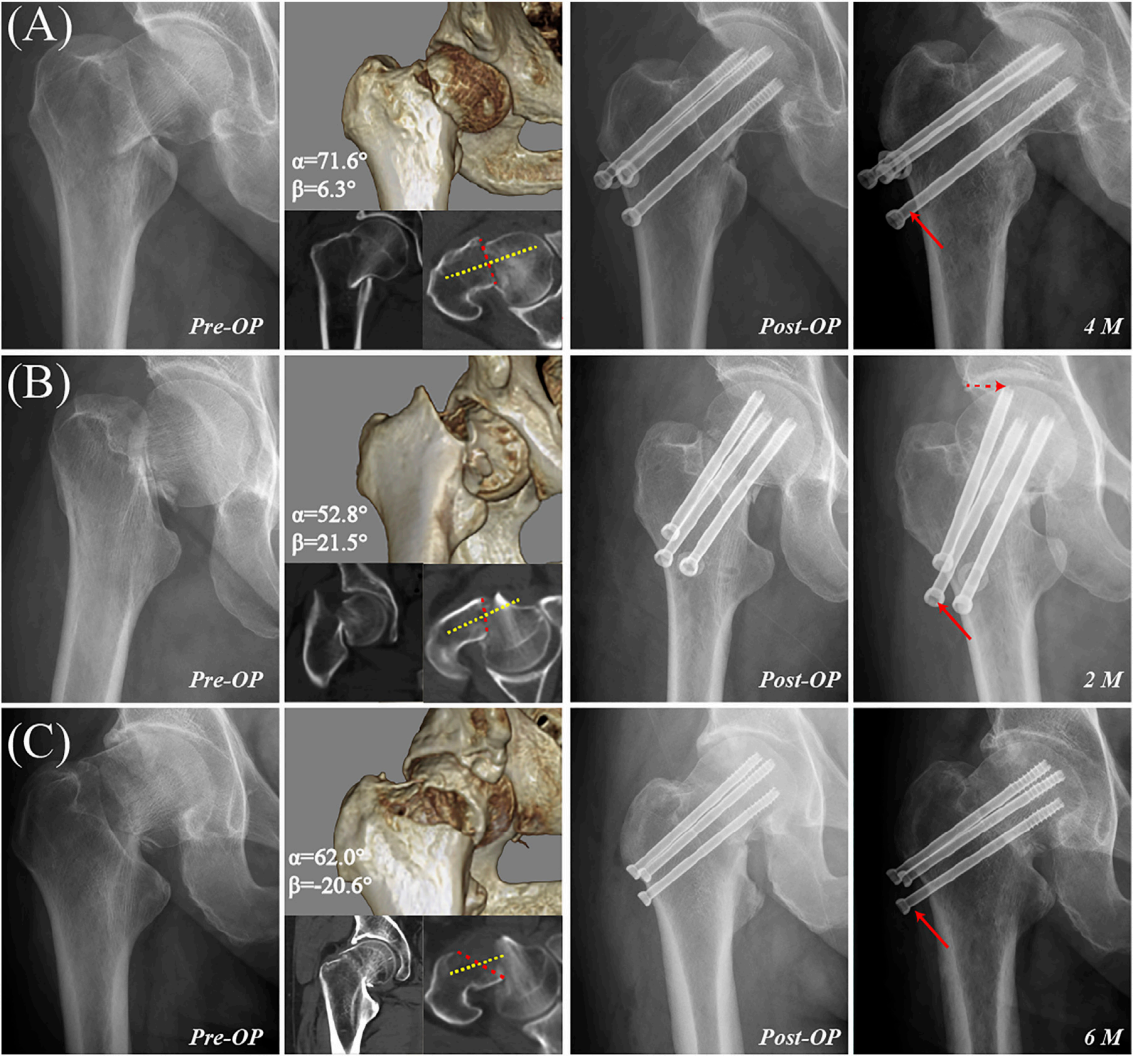
Examples of femoral neck fractures with 3-D unstable inclination angle. **(A)** A 46-year-old male with a vertical unstable inclination angle (α = 71.6° and β = 6.3°) was treated with three cannulated screws. Radiography taken 4 months postoperatively revealed severe screw withdrawal (red arrow) as well as femoral neck shortening, varus collapse, and delayed union. **(B)** A 46-year-old female with retroversion oblique unstable inclination angle (α = 52.8° and β = 21.5°) was treated by three cannulated screws with a fair reduction quality. Radiography taken 2 months postoperatively revealed severe screw withdrawal (solid red arrow) as well as femoral neck shortening, varus collapse, screw penetration of the joint capsule (dotted red arrow), and delayed union. **(C)** A 59-year-old male with anteversion oblique unstable inclination angle (α = 62.0° and β = −20.6°) was treated with three cannulated screws. Radiography taken 6 months postoperatively revealed severe screw withdrawal (red arrow) as well as femoral neck shortening, varus collapse, and delayed union.

**FIGURE 6:**
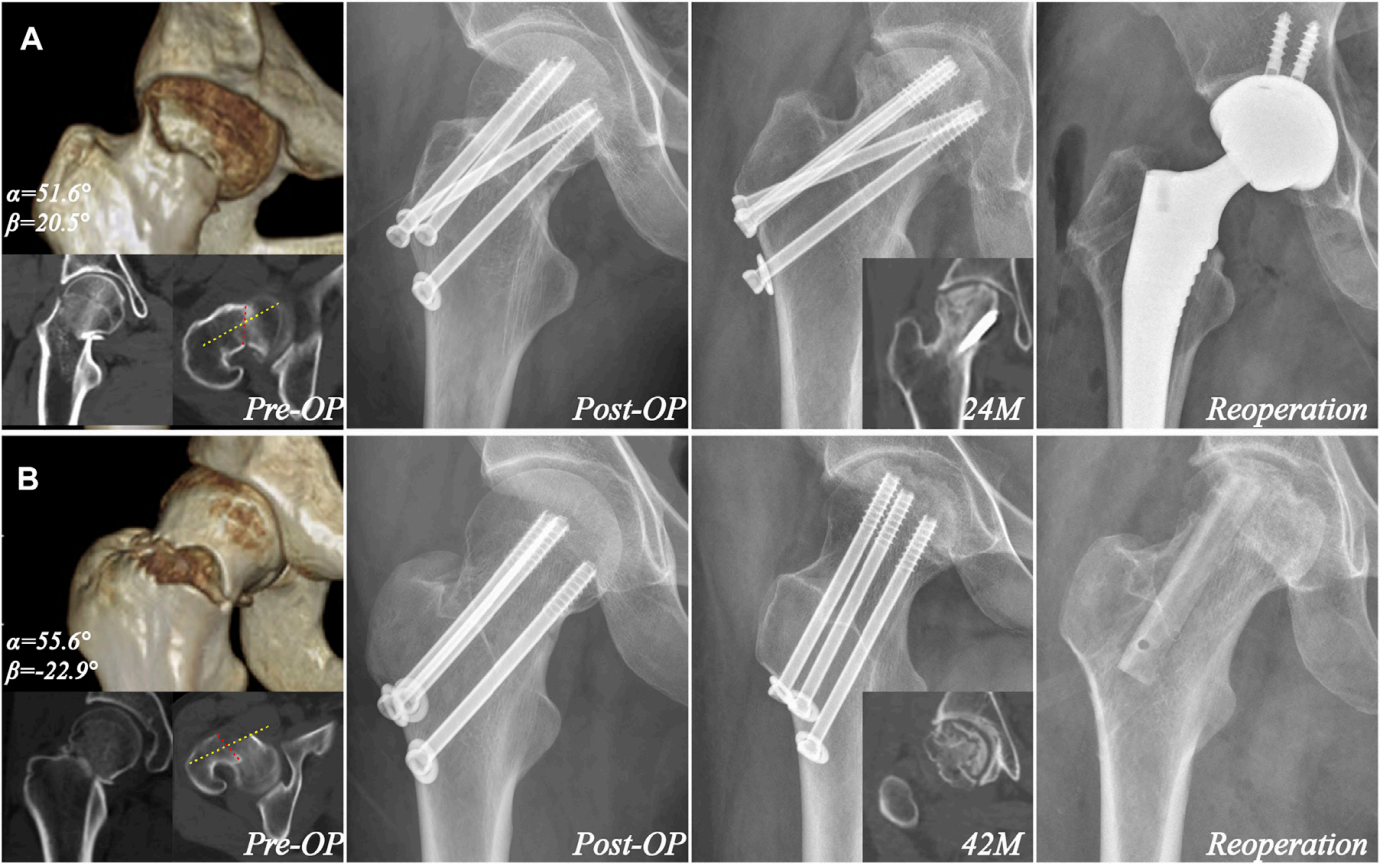
Examples of avascular necrosis in cases with oblique unstable inclination. **(A)** A 42-year-old female with retroversion oblique unstable inclination angle (α = 51.6° and β = 20.5°) was treated with four cannulated screws. Radiography taken 24 months postoperatively revealed avascular necrosis, which was treated with arthroplasty. The yellow and red line showed the femoral neck axis and fracture orientation in one CT plane. **(B)** A 32-year-old female with anteversion oblique unstable inclination angle (α = 55.6° and β = −22.9°) was treated with three cannulated screws. Radiography taken 42 months postoperatively revealed avascular necrosis, which was treated with free vascularized fibular grafting. The yellow and red line showed the femoral neck axis and fracture orientation in CT plane.

Binary logistic regression models indicated that reoperation was significantly associated with fracture’s 3-D inclination angle (OR = 4.699 (95%CI 2.949–7.486), p-value <0.001). The reoperation rate in 3-D unstable group (32.7%) is significantly (*p* < 0.001) higher than that in 3-D stable group (7.9%). Apart from reoperation risks, complications including non-union, femoral neck shortening and avascular necrosis were all significantly lower (p-value <0.05) in fractures designated as 3-D unstable inclinations (Figures 5, 6) (Table 2).

**TABLE 2:** Comparison of complications between 3-D stable and unstable groups.

	3-D stable	3-D unstable	*p*
N	593	162	—
Reoperation	47 (7.9%)	53 (32.7%)	<0.001
Non-union	23 (3.8%)	13 (8.02%)	0.028
Femoral neck shortening	86 (14.5%)	51 (31.5%)	<0.001
Avascular necrosis	113 (19.1%)	46 (28.4%)	0.010
Complication	158 (23.8%)	67 (45.1%)	0.008

**FIGURE 7:**
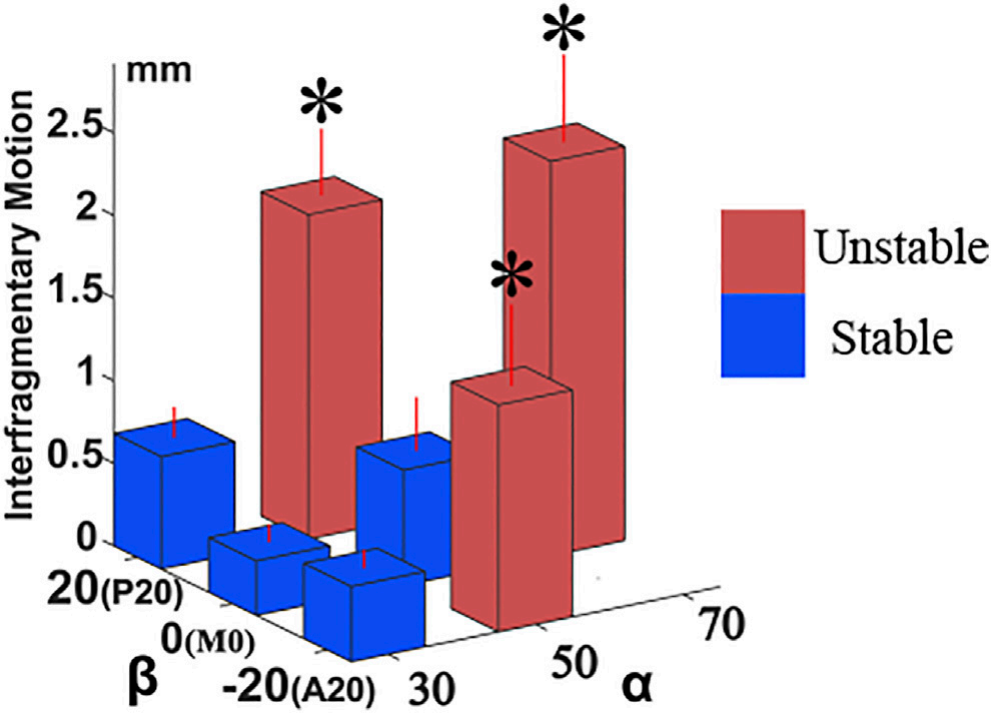
The comparison of interfragmentary motion in biomechanical test. IFM plots as a function of α and *β* angles in the biomechanical validation test. Synbone^®^ fracture groups with combinations of α = 70°; α = 50° and β = 20°; and α = 50° and β = 20° had significantly greater IFMs than groups with combinations of α = 30° (<50°), and α = 50° and β = 0°. These outcomes of simulated FNFs in Synbones^®^ correspond well with clinical findings. Asterisks indicate significance as evaluated by one-way ANOVA. Extent of group (*n* = 5) variability is standard deviation (SD) shown by red-colored vertical lines.

In the combined biomechanical validation test (Figure 7), the IFM was significantly greater (*p* < 0.05) in the 70M0 (*α* = 70°, *β* = 0°; 2.387 ± 0.532 mm); 50P20 (*α* = 50°, *β* = 20°; 1.963 ± 0.406 mm); and 50A20 (*α* = 50°, *β* = −20°; 1.374 ± 0.491 mm) groups than that in any other group of α and *β* combinations. The IFMs of the remaining groups (50M0 (*α* = 50°, *β* = 0°; 0.699 ± 0.327 mm), 30M0 (*α* = 30°, *β* = 0°; 0.330 ± 0.184 mm), 30A20 (*α* = 30°, *β* = −20°; 0.456 ± 0.104 mm), and 30P20 (*α* = 30°, *β* = 20°; 0.681 ± 0.184 mm) were not significantly different.

## Discussion

In the present study, we calculated 3-D fracture inclinations of a comprehensive set of FNFs in 755 patients using 3-D digitally reconstructed models from CT scans. We analysed and identified unstable types of 3-D inclinations based on the patient’s clinical prognosis, and validated these types in a biomechanical experiment. Thereby, the effect of fracture inclination in both vertical and oblique planes were firstly clarified in the current study. The 3-D evaluating method presented as a useful complement to the classic Pauwels method. Fractures with *α* > 70°, or 50°<*α* < 70° and *β* > 20°/*β* < -20° were real unstable fracture types in 3-D view, with significantly higher IFM and higher reoperation and complication risks.

With the 3-D inclination measurement, apart from vertical angulation, severe fracture obliquity axially (*β* > 20° or *β* < −20°) was observed in 24.11% patients. Variability in axial obliquity may be related to a combination of shear and rotational forces (Augat et al., 2019), patients experienced upon the traumatic impact and the position of the lower limb during the impact. Previous studies have confirmed that fracture obliquity reduces fracture stability (Collinge C A et al., 2014; ;Wright et al., 2020), but the concept of an oblique instability, has not been routinely considered in current preoperative evaluations. The present study is the first to quantitatively illustrate the influence of 3-D inclinations in both of vertical and oblique planes on fracture stability and clinical prognosis.

What is an “unstable” fracture after fixation? It can be defined as a fracture that has a large motion of the two fragments at the fracture site when under physiological load (An, 2018), even with anatomic reduction and standard internal fixation. Therefore, compared with construct stability, interfragmentary stability is more consistent with clinical prognosis (Jiang et al., 2021a). In this study, fractures with 3-D inclination angles having *α* > 70°; 50°<*α* < 70° and *β* > 20°/*β* < -20° should be demonstrated as the real unstable types because of significantly greater IFM than other *α* and *β* angle combinations of fractures assessed in the biomechanical test. Most of the unstable fractures with *α* > 70° were the “centered” type (i.e., −20°<*β* < 20°), indicating that they were likely the result of predominantly violent vertical forces, while those with 50°<α < 70° and *β* > 20°/*β* < -20° were produced by a combination of vertical and rotational forces (Augat et al., 2019). Fracture obliquity affected stability only to a certain extent. Either anteversion or retroversion over 20° significantly reduced stability in fractures with 50°<α < 70°, while no such effect on stability was observed in fractures with α < 50°.

Higher rates of non-union, femoral neck shortening and avascular necrosis resulted in a higher reoperation rate in 3-D unstable fractures. Excess IFM, especially in the shear direction, may lead to osteoclastogenesis (Ma et al., 2019) and bone resorption, which may eventually result in femoral neck shortening (Zlowodzki et al., 2008). Severe shortening of the femoral neck will lead to abductor moment reduction and irritation from protruding screws, which significantly decreases functional scores of patients (Slobogean et al., 2017; Felton et al., 2019). In the present study, there was a significantly (*p* = 0.02) higher incidence of avascular necrosis in fractures with 50°<α < 70° and *β* > 20°/*β* < -20° (See Supplementary Table S2), probably due to a difficulty of vessel reconstruction in these fractures.

Identifying the exact fracture type of a femoral neck fracture is prerequisite to develop appropriate treatment and rehabilitation plans, which can maximally reduce the potentially high risks of complications, and thus halt or delay the progression to arthroplasty. In clinical practice, surgeons should be aware of the inherent high risks of complications in fractures with unstable inclination angles, and handle these patients more prudently and schedule closer follow-ups. Because of the unstable nature, apart from achieving an acceptable reduction, a more biomechanically beneficial fixation strategy such as sliding hip screws (Bhandari and Swiontkowski, 2017; Augat et al., 2019), Buttress plate (Zhan et al., 2020; Jiang et al., 2021c), Alpha fixation (Jiang et al., 2021b; Jiang et al., 2021c) or the Tragon locking plate system (Bliven et al., 2020) were more recommended for these fractures compared with traditional three parallel screws. However, clinical evidence of the optimal internal fixation selection for the real unstable femoral neck fractures is still absent. Current available clinical studies such as the FAITH trail (Slobogean et al., 2017) all evaluated fracture stability according to Pauwels classification. The Pauwels angle based on 2-D radiography cannot reflect the real vertical inclination angle, which is subjective and inconsistent among observers (Embden et al., 2011). Therefore, further multicentric prospective or randomized controlled trials are still required to clarify the optimal internal fixation selection for the unstable femoral neck fractures identified in the current prognostic study. Finally, due to the tendency of re-displacement postoperatively in these fractures, a relatively conservative rehabilitation plan such as prolonging the time in bed or postponing weight-bearing exercise is preferred.

The current study had some limitations. Currently, CT scan is not a clinical routine, and there is no internationally uniform standard for identifying to whom CT scan should apply. Whether the cases in our hospital received CT scans is determined by the surgeon’s own experience, rather than patient’s specific condition or fracture severity, but a potential selection bias may still exist in the analysis. Although the 3-D inclination angle of FNFs showed a close association with reoperation outcome, the practical measurement of 3-D inclination angle needs to be simplified for convenience in order to be applied routinely in clinical applications. Nevertheless, the measurement of 3-D inclination angle is not beyond the abilities of most modern radiology departments, and given the potential long-term benefits, may well be worth the effort. Additionally, this study mainly concentrated on fracture inclination. Fracture comminution is another important factor determining interfragmentary stability, which should be analysed as a separate topic in future studies.

Internal fixation for femoral neck fractures of nongeriatric patients poses a clinical challenge, one with dissimilar prognoses for different fracture types. Evaluating fracture stability from both vertical and oblique planes in 3-D view could be a useful complement to the classic Pauwels method. Apart from vertical inclination, severe obliquity (*β* > 20°/*β* < -20°) was observed in 24.11% of all cases, a previously underappreciated rate. FNFs with particular combinations of α and β angles (*α* > 70°; 50°<α < 70° and *β* > 20°/*β* < −20°) produce worse fracture stability, higher complication rate, and reoperation risks. For these unstable FNFs, fixation with traditional three parallel screws showed a significantly greater interfragmentary motion across fracture site, thus a more mechanically stable internal fixation and conservative rehabilitation schedule are required in order to achieve a successful treatment outcome, improve life quality and reduce the burdensome healthcare costs of treating complications after suboptimal treatment.

## Data Availability

The raw data supporting the conclusion of this article will be made available by the authors, without undue reservation.
