# Supplementary material for: The Effect of Vertical and Oblique Inclinations on Fracture Stability and Reoperation Risks in Femoral-Neck Fractures of Nongeriatric Patient
Source: Front Bioeng Biotechnol. 2021 Nov 3;9:782001. doi: 10.3389/fbioe.2021.782001 (PMC8595327; doi:10.3389/fbioe.2021.782001)
Supplement: Supplementary file 1 [file Table1.DOCX]

Supplementary Material

**Supplementary Table1**

**Reoperation Reasons for the Included Patients (N=137)**

| **Type of Reoperation** | | N |
| --- | --- | --- |
| Removal of implants (N=58) | Fixation failure | 21 |
|  | Patient request | 37 |
| Secondary hip arthroplasty | | 65 |
| Vascularized fibular grafting | | 10 |
| Re-osteosynthesis | | 4 |
| **Total relevant reoperations** | | **100** |
| Total reoperations | | 137 |

Among the 755 included patients, 137 patients had reoperations within 5 years. Only participant data of relevant reoperations (n=100) were included in the analysis. We believed that this was reasonable, because a number of patients (n=37) requested that their implant be removed, even though they had no complications. These latter cases were excluded in this study, because they had no predictive value in terms of the 3-D parameters we were using. The reason for relevant reoperation included non-union (7 with arthroplasty, 4 with re-osteosynthesis, 1 with vascularized fibular grafting), femoral neck necrosis (58 with arthroplasty, 9 with vascularized fibular grafting) and screw withdrawal or protruding (21 with Removal of implants).

**Supplementary Table2**

**Sub-analysis of fractures with different 3-D inclination angles**

|  | **3-D Stable** | | | **3-D Unstable** | | |
| --- | --- | --- | --- | --- | --- | --- |
|  | Pauwels | Pauwels | P-value | Vertical Unstable | Oblique Unstable | P-value |
|  | Unstable | Stable |  |  |  |  |
| N | 355 | 238 |  | 69 | 93 |  |
| Reoperation | 26 (7.3%) | 20 (8.4%) | 0.630 | 18 (26.1%) | 36 (38.7%) | 0.092 |
| Non-union | 15 (4.2%) | 8 (3.4%) | 0.593 | 5 (7.3%) | 8 (8.6%) | 0.753 |
| Femoral Neck shortening | 56 (15.8%) | 30 (12.6%) | 0.283 | 23 (33.3%) | 28 (30.1%) | 0.662 |
| Avascular Necrosis | 70 (19.7%) | 43 (18.1%) | 0.616 | 13 (18.9%) | 33 (35.5%) | **0.020*** |
| Complication | 100 (28.2%) | 58 (24.4%) | 0.305 | 26 (37.7%) | 41 (44.1%) | 0.413 |

Vertical Unstable group: Fractures with α>70°;

Oblique Unstable group: Fractures with 50°<α<70° and β>20° or <-20°;

Pauwels Unstable group: Fractures with 50°<α<70° and -20°<β<20°, which are categorized as classic Pauwels Type Ⅲ fractures;

Pauwels Stable group: Fractures with α<50°, which are categorized as classic Pauwels Type Ⅰ/Ⅱ fractures
